# Zwitterionic Polypeptoids: A Promising Class of Antifouling Bioinspired Materials

**DOI:** 10.3390/ma15134498

**Published:** 2022-06-26

**Authors:** Jian Ding, Xiangmin Ding, Jing Sun

**Affiliations:** 1 College of Polymer Science and Engineering, Qingdao University of Science and Technology, Qingdao 266042, China; jianding1996@163.com (J.D.); minding1997@163.com (X.D.); 2 State Key Laboratory of Supramolecular Structure and Materials College of Chemistry Jilin University, Changchun 130012, China

**Keywords:** zwitterionic polymer, polypeptoid, ring-opening polymerization, antifouling hydrogel, nanoparticle

## Abstract

Biofouling caused by protein adsorption and microbial colonization remains a great challenge in many applications. In this work, we synthesized a new type of zwitterionic polypeptoid containing carboxybetaine (CB) moieties (PeptoidCB) through thiol–ene chemistry of poly(N-allylglycine) (PNAG). The zwitterionic antifouling hydrogel was subsequently prepared by co-mixing PeptoidCB with agarose, which exhibited excellent resistance to non-specific protein adsorption and bacterial adhesion. Further, PeptoidCB-modified block copolypeptoids with amphiphilic structure were synthesized to form nanoparticles in an aqueous solution with neglected protein adsorption. The ability of PeptoidCB to resist non-specific protein adsorption and bacterial adhesion makes it a promising candidate for biomedical and industrial applications.

## 1. Introduction

Biofouling, such as undesirable protein adsorption, microorganism adhesion, and biofilm formation, remains a vital issue for applications of biomedicine and bioengineering [1]. For example, protein adsorptions onto the surface of biological implants, such as catheters and prosthetic devices, reduce the device efficacy and lead to thrombosis, hemolysis, and immune responses etc., [2,3,4]. Non-specific protein adsorption can also compromise the diagnostic accuracy [5]. Accidentally adhered bacteria can colonize on a surface, which causes the biofilm formation [6,7]. As a result, serious infections may occur and threaten the life of the patient [8]. Additionally, biofouling also emerges as a serious hazard to marine navigation and membrane manufacture [9].

In order to address the biofouling problem, various polymers have been developed to construct a hydrophilic surface, such as poly(ethylene glycol) (PEG), to reduce biofouling [6,10]. PEG is considered as the gold standard for unfouling polymers due to its excellent biocompatibility [11]. However, it was reported that PEG has poor stability and has induced antibodies after repeated usage, which has resulted in great limitations for biological applications [12,13]. Zwitterionic polymer, an excellent alternative to PEG, shows great potential in antifouling applications to prevent protein and bacterial adsorptions [14,15,16,17,18]. Zwitterionic polymers possess an equal number of uniformly distributed cations and anions along their polymer chains, which give rise to ultra-hydrophilicity [19,20]. Particularly, carboxybetaine (CB)-based polymers show superior advantages, such as excellent antifouling properties and biocompatibility, as compared with sulfobetaine (SB)- and phosphorylcholine (PC)-based zwitterionic polymers [21]. Recently, bioinspired polymers with zwitterionic pendant motifs on the peptidomimetic backbone have been receiving increasing attention [22,23]. In particular, polypeptoids, a class of nitrogen-substituted polyglycines, have emerged as promising peptidomimetic polymers with excellent biocompatibility and bioactivities [24,25,26]. They differ from polypeptides in their lack of hydrogen-bonding and chirality on the backbone, which offers a highly tunable structure–property relationship [27]. Herein, we have synthesized a new type of zwitterionic polypeptoid containing CB moieties (PeptoidCB) through thiol–ene chemistry of poly(*N*-allylglycine) (PNAG). The zwitterionic antifouling hydrogel was subsequently prepared by simply mixing the zwitterionic polypeptoid with the agarose, which exhibit excellent resistance to non-specific protein adsorption and bacterial adhesion. Furthermore, the CB-modified block copolypeptoid (PeptoidCB-2) with an amphiphilic chemical structure were synthesized in order to construct assemblies in an aqueous solution. We demonstrated that the obtained nanoparticles can barely absorb the proteins. All of these results suggest that the obtained zwitterionic PeptoidCB show great potential for antifouling applications.

## 2. Materials and Methods

### 2.1. Materials and Instruments

Hexane and tetrahydrofuran (THF) were purified by passing them through activated alumina columns. Dichloromethane (CH_2_Cl_2_) was stored over calcium hydride (CaH_2_) and purified by reduced pressure distillation. Glyoxylic acid monohydrate, benzylamine (Bn-NH_2_), allylamine, and octylamine were obtained from Sigma-Aldrich Reagent Co., Ltd. DL-dithiothreitol (DTT), 2,2-dimethoxy-2-phenylacetophenone (DMPA), and fluorescein isothiocyanate (FITC) were purchased from Aladdin Reagent. Triethylamine and phosphorus trichloride were obtained from Sinopharm Chemical Reagent Co., Ltd. Bovine serum albumin (BSA) and *Staphylococcus aureus* (*S. aureus*) (ATCC6538) were obtained from Qingdao Hope Bio-Technology Co., Ltd. All other chemicals were obtained from commercial suppliers and used directly. ^1^HNMR measurements were performed on a Bruker AV500 FT-NMR spectrometer. Gel permeation chromatography (GPC) analysis was performed using an SSI pump to Wyatt Optilab DSP and RID-10A refractive index detector with 0.02 M LiBr in DMF as the eluent at a flow rate of 1.0 mL/min at 50 ℃. All GPC samples were prepared with concentrations of around 5 mg/mL. The molecular weights were calibrated against polystyrene (PS) standards. Atomic force microscopy (AFM) studies were conducted using tapping mode AFM (Bruker Multimode 8 AFM/SPM system) in ambient air with Nanoscope software. A total of 5 μL of polymer solution was placed on the mica by spin-coater and dried on freshly cleaved mica under ambient conditions before AFM imaging.

### 2.2. Synthesis of 2-Ethoxy-N-(2-mercaptoethyl)-N,N-dimethyl-2-oxoethan-1-aminium Bromide

Bis(2-dimethylaminoethyl)disulfide dihydrochloride (3.7 g, 13.2 mmol) and NaOH (1.1 g, 27.5 mmol) were dissolved in 30 mL of deionized (DI) water with stirring for 6 h at room temperature. The free base bis [2-(*N*,*N*-dimethylamino)ethyl] disulfide was then extracted with dichloromethane (3 × 100 mL), dried, and concentrated in vacuo. Bis [2-(*N*,*N*-dimethylamino)ethyl] disulfide (2.7 g, 13.0 mmol) was dissolved in 40 mL of acetonitrile solution, then ethyl bromoacetate (4.3 mL, 38.9 mmol) was added dropwise and stirred at room temperature for one hour. The precipitates were filtered, washed with acetonitrile and ether, followed by drying in vacuo. The obtained product (2.5 g, 4.6 mmol) and DTT (0.8 g, 5.2 mmol) were dissolved in 30 mL methanol and stirred for 12 h. The reaction solution was concentrated and precipitated in cold ether. The precipitates were further washed with diethyl ether, filtered, and dried in vacuum to obtain white powder (79.8% yield).

### 2.3. Synthesis of Poly(N-allylglycine) (PNAG) Homopolymers

The *N*-allyl *N*-carboxyanhydride (Allyl-NCA, M_1_) and *N*-octyl *N*-carboxyanhydride (Oct-NCA, M_2_) monomers were synthesized according to a previous work [28]. Typically, *N*-allyl *N*-carboxyanhydride (Allyl-NCA, M_1_) (1 g, 7.1 mmol) was dissolved in anhydrous tetrahydrofuran in a glovebox (100 mg mL^−1^) under a nitrogen atmosphere. Anhydrous benzylamine in tetrahydrofuran, [M_1_]_0_/[−NH_2_]_0_ = 50:1 was then added. The polymerization was carried out in a N_2_ atmosphere at 55 °C. The reaction was monitored by infrared spectroscopy, where the disappearance of characteristic peaks of NCA indicate the complete reaction. The solution was then precipitated in cold ether to prepare a white solid (71.2% yield). By changing the ratio of NCA/initiators (benzylamine), different degrees of polymerization were obtained in a similar way.

### 2.4. Synthesis of Poly(N-allylglycine)-b-Poly(N-octylglycine) Diblock Copolymers

In a similar way, as the reaction of *N*-allyl *N*-carboxyanhydride (Allyl-NCA, M_1_) (1 g, 7.1 mmol) was completed, *N*-octyl *N*-carboxyanhydride (Oct-NCA, M_2_) (0.3 g, 1.4 mmol) was added into the reaction system under a nitrogen atmosphere at 55 °C ([M_1_]_0_/[M_2_]_0_/[−NH_2_]_0_ = 50:10:1). The reaction was monitored by infrared spectroscopy, where the disappearance of characteristic peaks of NCA indicate the complete reaction. The solution was then precipitated in cold ether to achieve a white powder (69.2% yield). By changing the ratio of M_1_/M_2_/initiators (benzylamine), different degrees of polymerization were obtained in a similar way.

### 2.5. Synthesis of Polypeptoid Modified with Quaternary Amino Group (PeptoidQA)

Typically, PNAG_49_ (300.0 mg, 0.062 mmol), DMPA (38.8 mg, 0.15 mmol), and 2-ethoxy-*N*-(2-mercaptoethyl)-*N*,*N*-dimethyl-2-oxoethan-1-aminium bromide (1.9 g, 15.2 mmol) ([−SH]/[−C=C]/[DMPA] = 500/100/5) were dissolved in 10 mL DMF. The system was degassed and then irradiated upon UV light for 4 h. After dialysis for 3 days, the solution was lyophilized to obtain white solid PeptoidQA (68.0% yield).

### 2.6. Synthesis of Polypeptoid Modified with Carboxybetaine (PeptoidCB)

PeptoidQA (100 mg) and Amberlite IRN78 hydroxide resin (500 mg) were dissolved in 5 mL Millipore water and stirred at room temperature for 4 h. The system was filtered and lyophilized to yield final PeptoidCB product as a white solid (85% yield).

### 2.7. Preparation of the Zwitterionic Hydrogels

The agarose (30 mg) and PeptoidCB (30 mg) were dissolved in deionized water (2 mL) with heating at ~100 °C. After cooling, the hydrogels were cut into pieces.

### 2.8. Protein Adsorption

Hydrogel sheets (1 × 1 cm) were soaked in PBS for 12 h and then rinsed three times for two minutes each time. The hydrogel sheets were then immersed in 2 mL of FITC-labeled bovine serum protein solution at a concentration of 0.1 mg/mL for 1 h at 37 °C. The hydrogel sheets were gently rinsed with PBS to remove the non-adsorbed bovine serum albumin from the surface. The image of hydrogel surface was record by an Olympus ix73 fluorescence microscope. The experimental parameter settings were consistent for comparison.

The protein adsorptions (Lz, BSA and Fg) on hydrogel surfaces were determined using the micro-BCA assay. Hydrogel sheets (1 × 1 cm) were soaked in PBS for 12 h and washed three times for two minutes each time. The hydrogels were then immersed in 2 mL of protein solution at a concentration of 0.1 mg/mL and then incubated at 37 °C for 24 h. The surface was gently washed with PBS and placed separately in a 24-well plate containing 1 mL sodium dodecyl sulfate (2.0 wt%) in each well. After shaking for two hours at 37 °C, the hydrogels were sonicated for one hour to separate the surface-bound proteins. The resulting solution (100 μL) was placed in a 96-well plate, 100-μL BCA reagent was added, and was incubated at 60 °C for 1 h. Protein concentration was recorded at 560 nm on a microplate reader. The efficiency of protein resistance was calculated as follows:

$$\mathrm{protein}\;\mathrm{resistance}\;\left(\%\right)=\frac{\left({\mathrm{OD}}_{\mathrm{Control}}-{\mathrm{OD}}_{\mathrm{Surface}}\right)}{{\mathrm{OD}}_{\mathrm{Control}}}\times 100\%$$

### 2.9. Bacteria Surface Adhesion Test

Hydrogel sheets (1 × 1 cm) were soaked in PBS for 12 h, rinsed for two minutes, which was repeated three times. The hydrogels were then immersed in 1 mL of bacterial suspension (3× 10^5^ CFU/mL in MH) and incubated at 37 °C for 6 h. The hydrogels were washed with PBS, followed by staining by a LIVE/DEAD BacLight bacterial viability kit for 10 min. The image of the hydrogel surface was record by a confocal laser scanning microscope (CLSM).

### 2.10. Preparation of Antifouling Micelles

All samples were dispersed in 1 mL of PBS solution with a concentration of 0.5 mg/mL. A total of 1 mg BSA was added into the solution and was further incubated at 37 °C for 1 h prior to measurement. The morphology of the zwitterionic assemblies was performed by atomic force microscopy (AFM) and dynamic light scattering (DLS).

## 3. Results

**Scheme 1:**
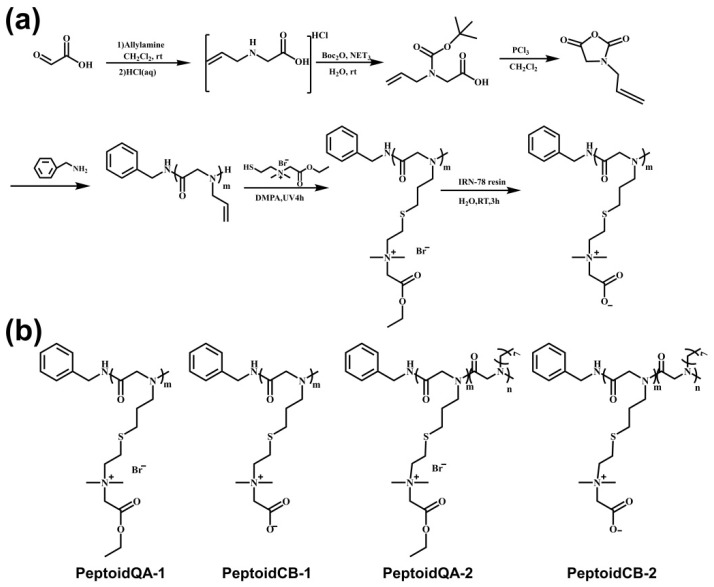
(**a**) Synthetic route to the zwitterionic polypeptoids, (**b**) chemical structures of CB-modified polypeptoids and QA-modified polypeptoids (1 represents homopolymer and 2 represents block copolymer).

**Figure 1:**
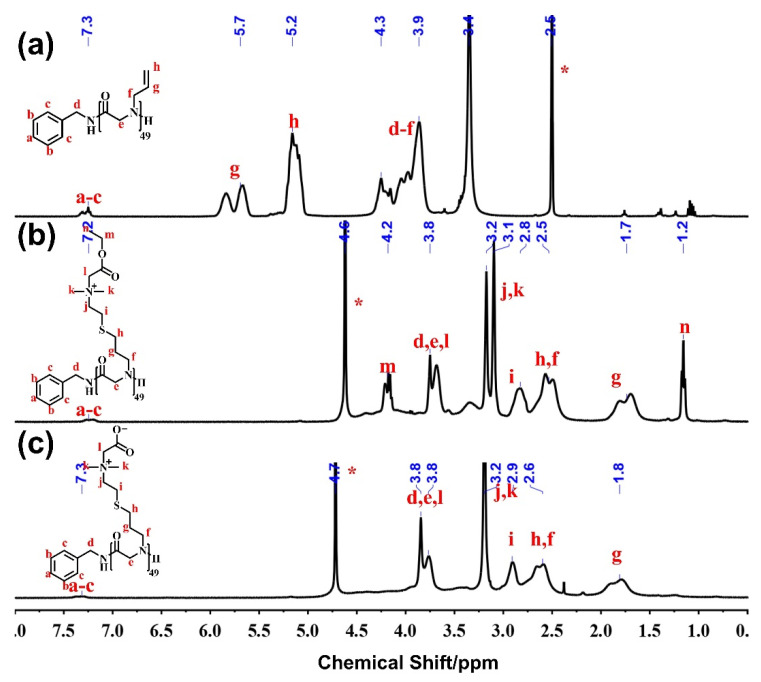
^1^H NMR spectra of CB-modified homopolypeptoids (**a**) PNAG, (**b**) PeptoidQA-1, and (**c**) Peptoid CB-1. * represents the solvent.

**Figure 2:**
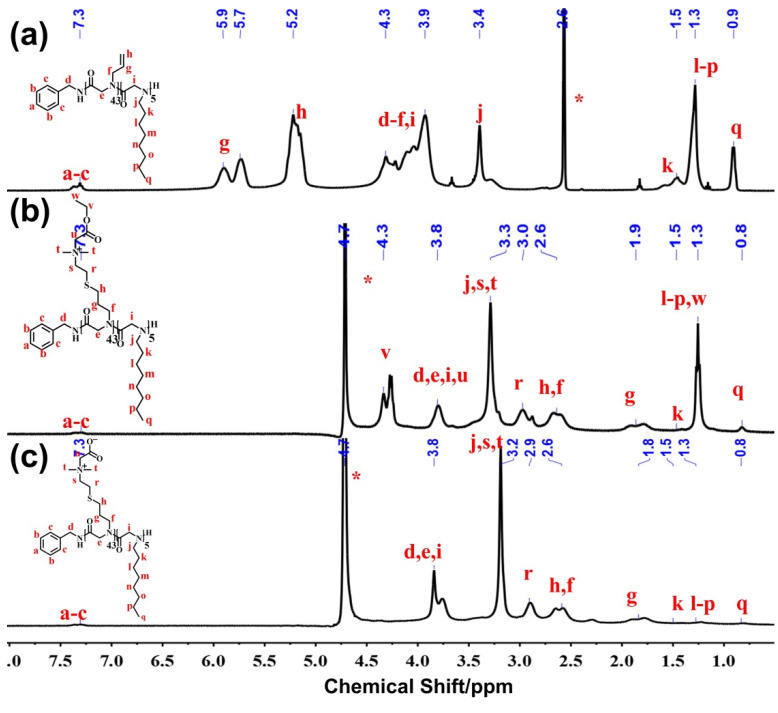
^1^H NMR spectra of CB-modified block copolypeptoids (**a**) PNAG-*b*-PNOG, (**b**) Peptoid QA-2, and (**c**) Peptoid CB-2. * represents the solvent.

The *N*-octyl-*N*-carboxyanhydride (Oct-NCA) and *N*-allyl-*N*-carboxyanhydride (Allyl-NCA) monomers were synthesized according to previously reported methods [28]. As show in Figure S1, the ^1^H NMR spectroscopy indicates the successful preparation of the monomer. The homopolymer (PNAG) and copolymers (PNAG-*b*-PNOG) were then synthesized by ring-opening polymerization (ROP) of Allyl-NCA and sequential ROP of Allyl-NCA and Oct-NCA using benzylamine (Bn-NH_2_) as the initiator, respectively (Scheme 1). The reaction was monitored by infrared spectroscopy, where the disappearance of characteristic *ν_C_*_=O_ peaks of NCA at 1790 and 1850 cm^−1^ indicate the complete reaction (Figure S2). By changing the different ratios of monomer/initiator, a series of polymers with different degrees of polymerization (DP) were synthesized, which was determined by ^1^H NMR spectroscopy. Figure 1a shows that all of the peaks are well assigned in the ^1^H NMR spectra, confirming the chemical structures of the polymers. The GPC traces typically show a unimodal peak, indicative of narrow molecular weight distribution (Figure S3). In addition, Table 1 summarizes the characteristics and dispersity (*Đ*) of all of the samples, where the subscripts m and n correspond to the DP_S__._ The polypeptoids were then modified with 2-ethoxy-*N*-(2-mercaptoethyl)-*N*,*N*-dimethyl-2-oxoethan-1-aminium (Figure S4). As show in Figure 1, the protons of the allyl group at 5.0–6.0 ppm completely disappear. Additionally, the protons of the end-ethyl group at 4.0–4.4 ppm and 1.16 ppm appear, indicative of the complete modification of functional groups. These results suggest that the quaternary amine group (QA) modified polypeptoid (PeptoidQA-1) is successfully prepared (Figure 1b). The carboxybetaine (CB) modified polypeptoid (PeptoidCB-1) was obtained after deprotection. The protons at 1.2 ppm and 4.5 ppm completely disappear, indicating the complete abscission of the terminal group (Figure 1c). Similarly, the QA-modified block copolymer (PeptoidQA-2) was prepared with the same protocol. After deprotection, the CB-modified polypeptoid (PeptoidCB-2) was prepared. The ^1^H NMR confirms the chemical structure with well-defined peaks (Figure 2).

Resistance to Protein Adsorption

**Figure 3:**
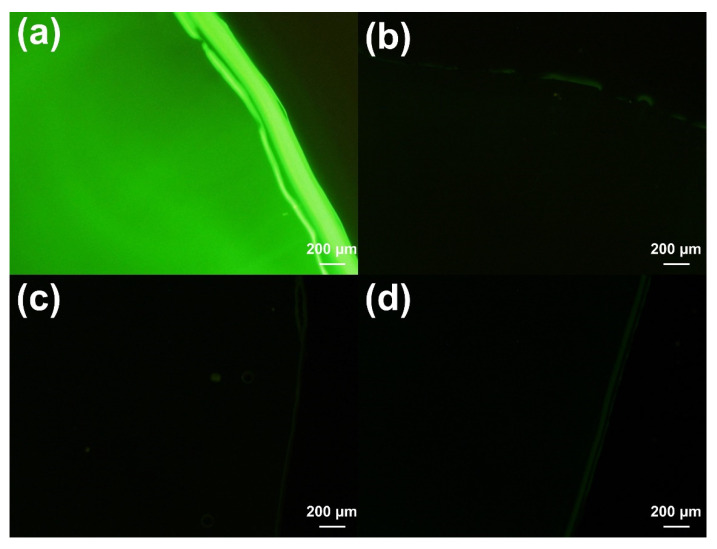
Fluorescence microscope images of (**a**) bare agarose hydrogel, (**b**) PeptoidCB_24_-1 hydrogel, (**c**) PeptoidCB_49_-1 hydrogel, (**d**) PeptoidCB_69_-1 hydrogel immersed in FITC-labeled BSA solution for 1 h.

In order to evaluate the adsorption of nonspecific proteins and bacterial adhesion properties of the zwitterionic polymers, we first prepared agarose hydrogels by mixing agarose with CB-modified homopolymer polypeptoid (PeptoidCB_m_-1) at different DPs. The activity of the hydrogel against protein fouling was assessed by using FITC-labeled bovine serum albumin (BSA) as a model protein, imaged by fluorescence microscopy. Figure 3 shows that fluorescence signals are barely observed in the hydrogels containing PeptoidCB_24_-1 (Figure 3b–d). Increasing the DP of the PeptoidCB_m_-1 yields similar results. In contrast, the control group of agarose hydrogel without PeptoidCB-1 shows an obvious fluorescence signal (Figure 3a). This indicates that a large amount of proteins are adsorbed on the surface of the control group. Instead, the PeptoidCB-1 hydrogel surface has significantly enhanced resistance to protein. We proposed that this is because the PeptoidCB-1 possesses a large number of positive and negative charges, which is overall neutral. This generates a hydration layer on the surface of the PeptoidCB-1 hydrogel via electrostatic interaction, which reduces the adsorption of proteins onto the surface of the hydrogel [29,30,31,32].

**Figure 4:**
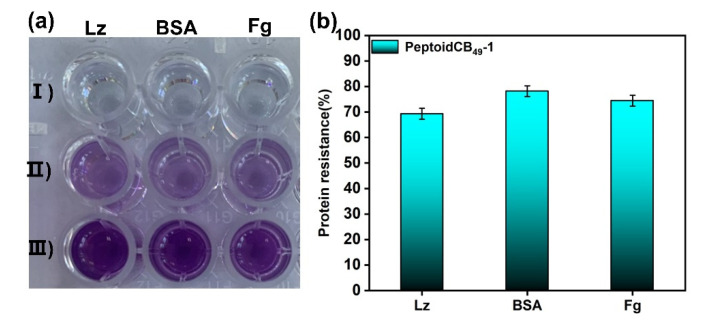
(**a**) Micro-BCA reagent (blank, I) chelated with the proteins from PeptoidCB_49_-1 hydrogel surface (II) and bare agarose hydrogel surface (III), (**b**) the resistance of different proteins (Lysozyme, BSA, and Fg) on the hydrogel surface (the error bars represent the standard deviation, n = 3).

In order to further quantify the adsorption performance of hydrogels with PeptoidCB-1, we evaluated the protein adsorption capacity with fibrinogen (Fg), bovine serum albumin (BSA), and lysozyme (Lz) by a micro-BCA protein assay [33]. The unabsorbed protein can chelate with the BCA regent and form a violet complex [23]. As shown in Figure 4a, the PeptoidCB_49_-1 hydrogel shows as light violet, as compared to the control group, which suggests that the protein adsorption on the surfaces with PeptoidCB_49_-1 is significantly reduced (Figure 4b). The protein resistance efficiencies were determined to be 74.5% (Fg), 78.2% (BSA), and 69.3% (Lysozyme), respectively. These results confirm the excellent antifouling properties of the PeptoidCB-1 hydrogels.

Resistance to Bacterial Adsorption

**Figure 5:**
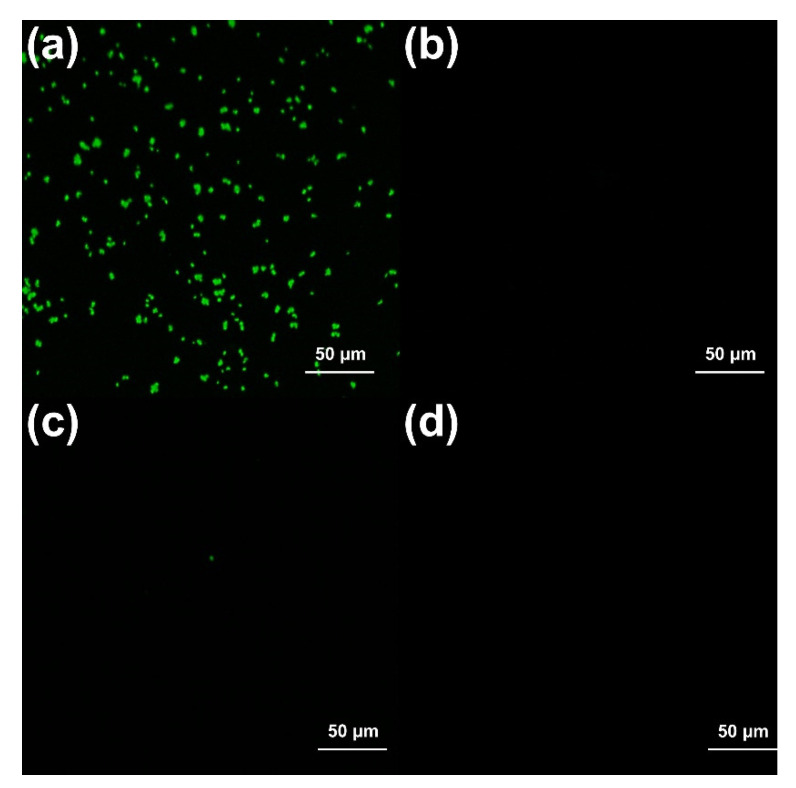
Confocal laser scanning microscope images of *S. aureus* accumulated on the (**a**) bare agarose hydrogel, (**b**) PeptoidCB_24_-1 hydrogel, (**c**) PeptoidCB_49_-1 hydrogel, and (**d**) PeptoidCB_69_-1 hydrogel.

The bacterial adhesion onto surfaces and subsequent formation of a biofilm are critical issues for many biomedical and engineering applications [1,34]. The resistance ability of PeptoidCB-1 hydrogels against bacterial adhesion was further evaluated by incubating *Staphylococcus aureus* (*S. aureus*) in PBS (3 × 10^5^ CFU mL^−1^) on the PeptoidCB-1 hydrogel. After incubation, the hydrogels were gently washed with sterile PBS solution in order to remove the loosely-bound bacteria from the surface. *S. aureus* cells were stained using live/dead bacterial activity kits and visualized under a confocal laser scanning microscope. Figure 5 shows over 2.5 × 10^5^ live *S. aureus* cell/mm^2^ with green dots were observed in the agarose hydrogel. In contrast, the green fluorescence on the PeptoidCB-1 hydrogels surface was barely observed, indicative of the presence of very few *S. aureus* cells (less than 100 live *S. aureus* cells/mm^2^). These results further confirm the superior antifouling property against the bacteria. We attributed this to strong hydration due to the electrostatic action on the PeptoidCB-1 hydrogels surface that reduces the unexpected adhesion of bacteria to the surface.

Self-assembly of the antifouling micelles

**Figure 6:**
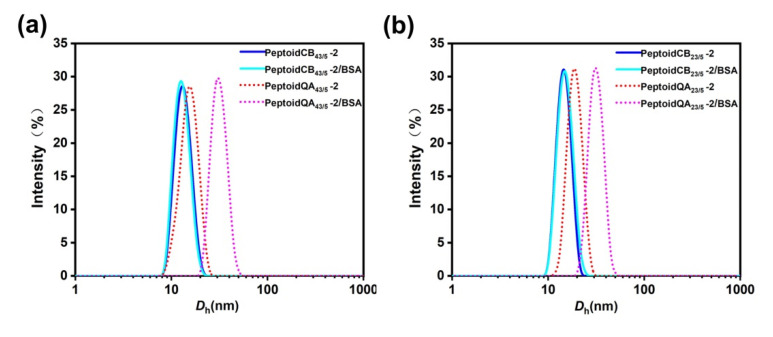
Interactions of BSA with (**a**) PeptoidCB_43/5_-2/PeptoidQA_43/5_-2 and (**b**) PeptoidCB_23/5_-2/PeptoidQA_23/5_-2 by DLS.

**Figure 7:**
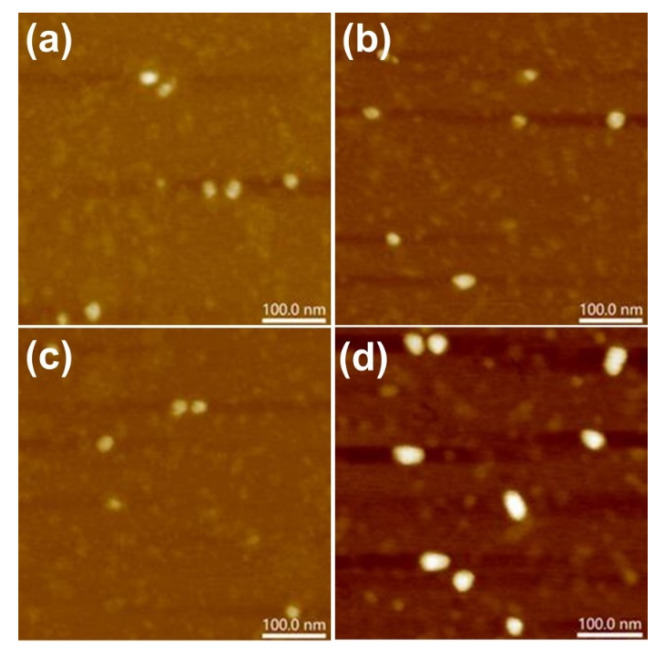
AFM images of (**a**) PeptoidCB_43/5_-2, (**b**) PeptoidCB_43/5_-2/BSA, (**c**) PeptoidQA_43/5_-2, and (**d**) PeptoidQA_43/5_-2/BSA.

The CB-modified block copolypeptoid (PeptoidCB-2) with amphiphilic chemical structure was synthesized in order to construct assemblies in an aqueous solution. For a direct comparison, both PeptoidCB-2 and PeptoidQA-2 were dispersed into PBS (0.5 mg/ mL), respectively. Both AFM and DLS were performed in order to study the morphology of the zwitterionic polymers. Figure 6 shows that the hydrodynamic diameters (*D*_h_) of the PeptoidQA_43/5_-2 assembly is determined to be 15 ± 1 nm, which is significantly smaller than that of the mixture with proteins (30 ± 2 nm). In contrast, the *D*_h_ of the PeptoidCB_43/5_-2 assembly remains consistent (13 ± 1 nm) before and after mixing with BSA. In addition, similar results are observed in the samples of PeptoidCB_23/5_-2 and PeptoidQA_23/5_-2 as well. The *D*_h_ of PeptoidCB_23/5_-2 remains quite similar (15 ± 1 nm) before and after mixing with proteins. However, an obvious increase from 18 ± 2 nm to 33 ± 2 nm was observed in the case of PeptoidQA_23/5_-2. The spherical morphology with a diameter of ~13 nm and ~15 nm were further observed for PeptoidCB_43/5_-2 and PeptoidQA_43/5_-2 by AFM, respectively (Figure 7a,c). After incubation with BSA, the size of the PeptoidCB_43/5_-2+BSA remained consistent, as indicated by the same diameter of ~13 nm (Figure 7b). This result suggests that the zwitterionic nanoparticles can barely absorb the proteins. In contrast, the diameter of the PeptoidQA_43/5_-2+BSA assembly increases to ~30 nm (Figure 7d). This is due to the presence of positively charged quaternary amine groups in PeptoidQA-2, which causes the adsorption of BSA on the surface of the spherical assembly.

## 4. Conclusions

In summary, we first prepared a series of poly(*N*-allylglycine) (PNAG) homopolymers and poly(*N*-allylglycine)-*b*-poly(*N*-octylglycine) block copolymers by ring-opening polymerization. The carboxybetaine-modified homopolymer polypeptoid (PeptoidCB-1) and the related diblock copolymer polypeptoid (PeptoidCB-2) were synthesized by thiol–ene chemistry. The hybrid hydrogels, produced by mixing agarose with PeptoidCB-1, exhibit excellent resistance ability to non-specific protein adsorption and unexpected bacterial adhesion. Further, the self-assemblies of PeptoidCB-2 also show remarkable resistance to protein adsorption. The preparation of PeptoidCB polymers with these excellent properties represent a new type of antifouling zwitterionic material.

## Figures and Tables

**Table 1:** Characteristics of the polymers synthesized.

Samples	Feed Ratio ^a^	DP ^b^	*M*n ^c^ (kDa)	*M*n ^d^ (kDa)	Dispersity (*Ð*) ^d^
PNAG_24_	1/30	24	2.43	2.04	1.08
PNAG_49_	1/50	49	4.86	4.23	1.04
PNAG_69_	1/70	69	6.80	6.21	1.14
PNAG_23_-*b*-PNOG_5_	1/30/10	23/5	3.18	2.83	1.17
PNAG_43_-*b*-PNOG_5_	1/50/10	43/5	5.12	4.76	1.20

^a^ Feed molar ratio of Bn-NH_2_/Allyl-NCA or Bn-NH_2_/Allyl-NCA/Oct-NCA, ^b^ calculated from ^1^H NMR spectra, ^c^ calculated from ^1^H NMR spectra, ^d^ determined from GPC.

## Data Availability

The data that support the findings of this study are available in the supplementary material of this article.

## Supplementary Materials

The following supporting information can be downloaded at the following address: https://www.mdpi.com/article/10.3390/ma15134498/s1, Figure S1: ^1^H NMR spectra of (a) Allyl-NCA in DMSO and (b) Oct-NCA (* indicates DMSO). Figure S2: FTIR spectra of Allyl-NCA and PNAG_49_. Figure S3: GPC chromatograms of the polymers. The molecular characteristics are shown in Table 1. Figure S4: ^1^H NMR spectra of 2-ethoxy-*N*-(2-mercaptoethyl)-*N*,*N*-dimethyl-2-oxoethan-1-aminium bromide.

## References

[1] Jiang S., Cao Z. (2010). Ultralow-Fouling, Functionalizable, and Hydrolyzable Zwitterionic Materials and Their Derivatives for Biological Applications. Adv. Mater..

[2] Franz S., Rammelt S., Scharnweber D., Simon J.C. (2011). Immune responses to implants—A review of the implications for the design of immunomodulatory biomaterials. Biomaterials.

[3] Amiji M., Park K. (1993). Surface modification of polymeric biomaterials with poly(ethylene oxide), albumin, and heparin for reduced thrombogenicity. J. Biomater. Sci. Polym. Ed..

[4] Venkatraman S., Boey F., Lao L.L. (2008). Implanted cardiovascular polymers: Natural, synthetic and bio-inspired. Prog. Polym. Sci..

[5] Hucknall A., Rangarajan S., Chilkoti A. (2009). In Pursuit of Zero: Polymer Brushes that Resist the Adsorption of Proteins. Adv. Mater..

[6] Cheng G., Zhang Z., Chen S., Bryers J.D., Jiang S. (2007). Inhibition of bacterial adhesion and biofilm formation on zwitterionic surfaces. Biomaterials.

[7] Cheng G., Li G., Xue H., Chen S., Bryers J.D., Jiang S. (2009). Zwitterionic carboxybetaine polymer surfaces and their resistance to long-term biofilm formation. Biomaterials.

[8] Kardela J.H., Millichamp I.S., Ferguson J., Parry A.L., Reynolds K.J., Aldred N., Clare A.S. (2019). Nonfreezable Water and Polymer Swelling Control the Marine Antifouling Performance of Polymers with Limited Hydrophilic Content. ACS Appl. Mater. Interfaces.

[9] Zhu M.-M., Fang Y., Chen Y.-C., Lei Y.-Q., Fang L.-F., Zhu B.-K., Matsuyama H. (2021). Antifouling and antibacterial behavior of membranes containing quaternary ammonium and zwitterionic polymers. J. Colloid Interface Sci.

[10] Ostuni E., Chapman R.G., Holmlin R.E., Takayama S., Whitesides G.M. (2001). A Survey of Structure−Property Relationships of Surfaces that Resist the Adsorption of Protein. Langmuir.

[11] Cao Z., Jiang S. (2012). Super-hydrophilic zwitterionic poly(carboxybetaine) and amphiphilic non-ionic poly(ethylene glycol) for stealth nanoparticles. Nano Today.

[12] Zhang L., Cao Z., Bai T., Carr L., Ella-Menye J.-R., Irvin C., Ratner B.D., Jiang S. (2013). Zwitterionic hydrogels implanted in mice resist the foreign-body reaction. Nat. Biotechnol..

[13] Shen M., Martinson L., Wagner M.S., Castner D.G., Ratner B.D., Horbett T.A. (2002). PEO-like plasma polymerized tetraglyme surface interactions with leukocytes and proteins: In vitro and in vivo studies. J. Biomater. Sci. Polym. Ed..

[14] Banerjee I., Pangule R.C., Kane R.S. (2011). Antifouling Coatings: Recent Developments in the Design of Surfaces That Prevent Fouling by Proteins, Bacteria, and Marine Organisms. Adv. Mater..

[15] Mi L., Jiang S. (2014). Integrated Antimicrobial and Nonfouling Zwitterionic Polymers. Angew. Chem. Int. Ed..

[16] Erathodiyil N., Chan H.-M., Wu H., Ying J.Y. (2020). Zwitterionic polymers and hydrogels for antibiofouling applications in implantable devices. Materials Today.

[17] Liu S., Tang J., Ji F., Lin W., Chen S. (2022). Recent Advances in Zwitterionic Hydrogels: Preparation, Property, and Biomedical Application. Gels.

[18] Asha A.B., Chen Y., Narain R. (2021). Bioinspired dopamine and zwitterionic polymers for non-fouling surface engineering. Chem. Soc. Rev..

[19] Shao Q., Jiang S. (2015). Molecular Understanding and Design of Zwitterionic Materials. Adv. Mater..

[20] Lowe A.B., McCormick C.L. (2002). Synthesis and Solution Properties of Zwitterionic Polymers. Chem. Rev..

[21] Ladd J., Zhang Z., Chen S., Hower J.C., Jiang S. (2008). Zwitterionic Polymers Exhibiting High Resistance to Nonspecific Protein Adsorption from Human Serum and Plasma. Biomacromolecules.

[22] Zhang P., Jain P., Tsao C., Yuan Z., Li W., Li B., Wu K., Hung H.-C., Lin X., Jiang S. (2018). Polypeptides with High Zwitterion Density for Safe and Effective Therapeutics. Angew. Chem. Int. Ed..

[23] Gao Q., Li X., Yu W., Jia F., Yao T., Jin Q., Ji J. (2020). Fabrication of Mixed-Charge Polypeptide Coating for Enhanced Hemocompatibility and Anti-Infective Effect. ACS Appl. Mater. Interfaces.

[24] Zhang D., Lahasky S.H., Guo L., Lee C.-U., Lavan M. (2012). Polypeptoid Materials: Current Status and Future Perspectives. Macromolecules.

[25] Gangloff N., Ulbricht J., Lorson T., Schlaad H., Luxenhofer R. (2016). Peptoids and Polypeptoids at the Frontier of Supra- and Macromolecular Engineering. Chem. Rev..

[26] Sun J., Zuckermann R.N. (2013). Peptoid Polymers: A Highly Designable Bioinspired Material. ACS Nano.

[27] Secker C., Robinson J.W., Schlaad H. (2015). Alkyne-X modification of polypeptoids. Eur. Polym. J..

[28] Ni Y., Sun J., Wei Y., Fu X., Zhu C., Li Z. (2017). Two-Dimensional Supramolecular Assemblies from pH-Responsive Poly(ethyl glycol)-b-poly(l-glutamic acid)-b-poly(N-octylglycine) Triblock Copolymer. Biomacromolecules.

[29] Huang H., Zhang C., Crisci R., Lu T., Hung H.-C., Sajib M.S.J., Sarker P., Ma J., Wei T., Jiang S. (2021). Strong Surface Hydration and Salt Resistant Mechanism of a New Nonfouling Zwitterionic Polymer Based on Protein Stabilizer TMAO. J. Am. Chem. Soc..

[30] Chen Z. (2022). Surface Hydration and Antifouling Activity of Zwitterionic Polymers. Langmuir.

[31] Chen S., Zheng J., Li L., Jiang S. (2005). Strong Resistance of Phosphorylcholine Self-Assembled Monolayers to Protein Adsorption:  Insights into Nonfouling Properties of Zwitterionic Materials. J. Am. Chem. Soc..

[32] He Y., Hower J., Chen S., Bernards M.T., Chang Y., Jiang S. (2008). Molecular Simulation Studies of Protein Interactions with Zwitterionic Phosphorylcholine Self-Assembled Monolayers in the Presence of Water. Langmuir.

[33] Lin X., Jain P., Wu K., Hong D., Hung H.-C., O’Kelly M.B., Li B., Zhang P., Yuan Z., Jiang S. (2019). Ultralow Fouling and Functionalizable Surface Chemistry Based on Zwitterionic Carboxybetaine Random Copolymers. Langmuir.

[34] Page K., Wilson M., Parkin I.P. (2009). Antimicrobial surfaces and their potential in reducing the role of the inanimate environment in the incidence of hospital-acquired infections. J. Mater. Chem..

