# Supplementary material for: Zwitterionic Polypeptoids: A Promising Class of Antifouling Bioinspired Materials
Source: Materials (Basel). 2022 Jun 26;15(13):4498. doi: 10.3390/ma15134498 (PMC9267628; doi:10.3390/ma15134498)
Supplement: Supplementary file 1 [file materials-15-04498-s001.zip › materials-1744029-supplementary.pdf]

Supporting Information

Zwitterionic Polypeptoids: A Promising Class of Antifouling Bioinspired Materials

Jian Ding [1,†], Xiangmin Ding [1,†] and Jing Sun [2,*]

[1] College of Polymer Science and Engineering, Qingdao University of Science and Technology, Qingdao 266042, China; jianding1996@163.com (J.D.); minding1997@163.com (X.D.)
[2] State Key Laboratory of Supramolecular Structure and Materials College of Chemistry Jilin University, Changchun 130012, China
* Correspondence: jingsun@jlu.edu.cn
† These authors contributed equally to this work.

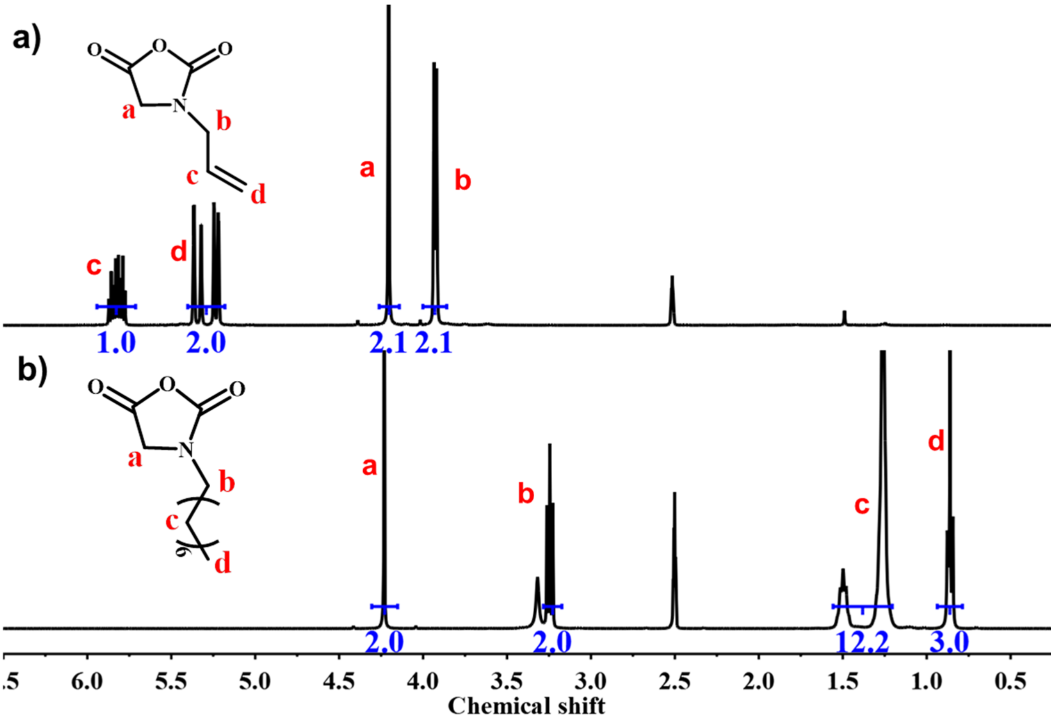

Figure S1. ^{1}H NMR spectra of (**a**) Allyl-NCA in DMSO and (**b**) Oct-NCA.

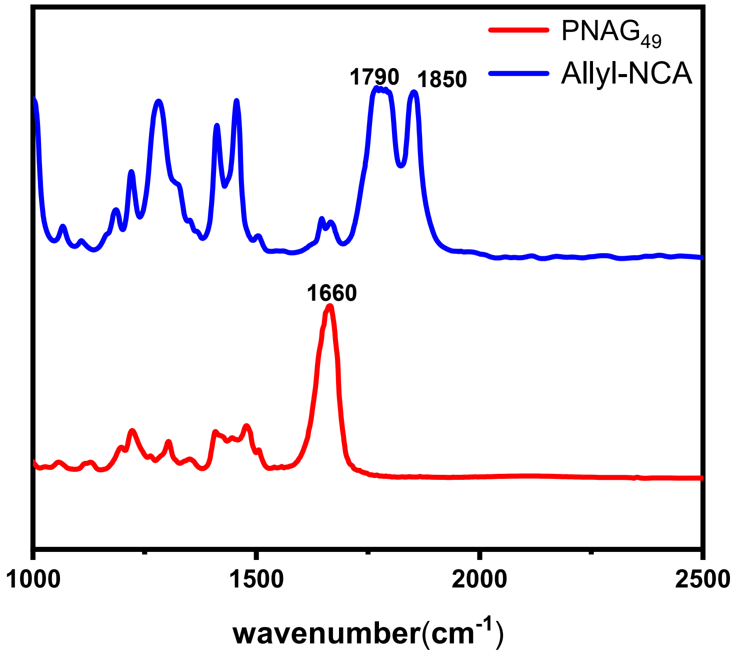

Figure S2. FTIR spectra of Allyl-NCA and $PNAG_{49}$.

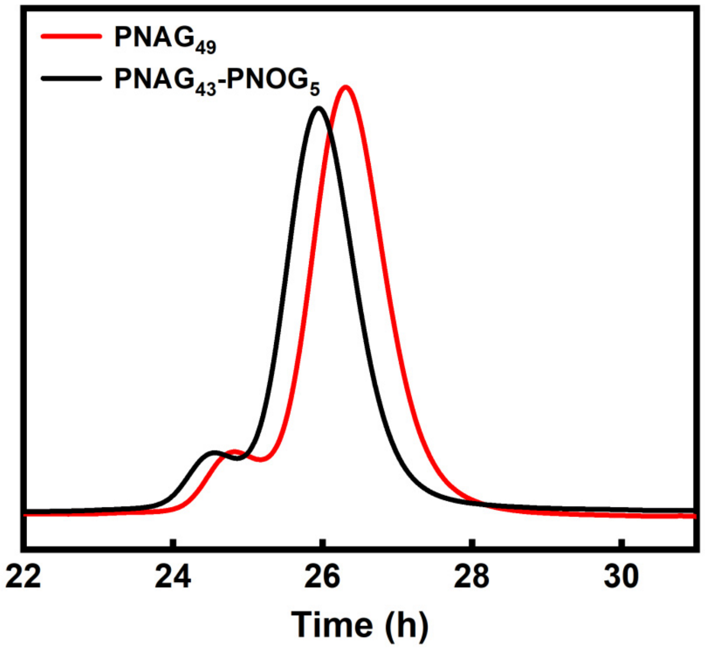

Figure S3. GPC chromatograms of the polymers. The molecular characteristics are shown in Table 1.

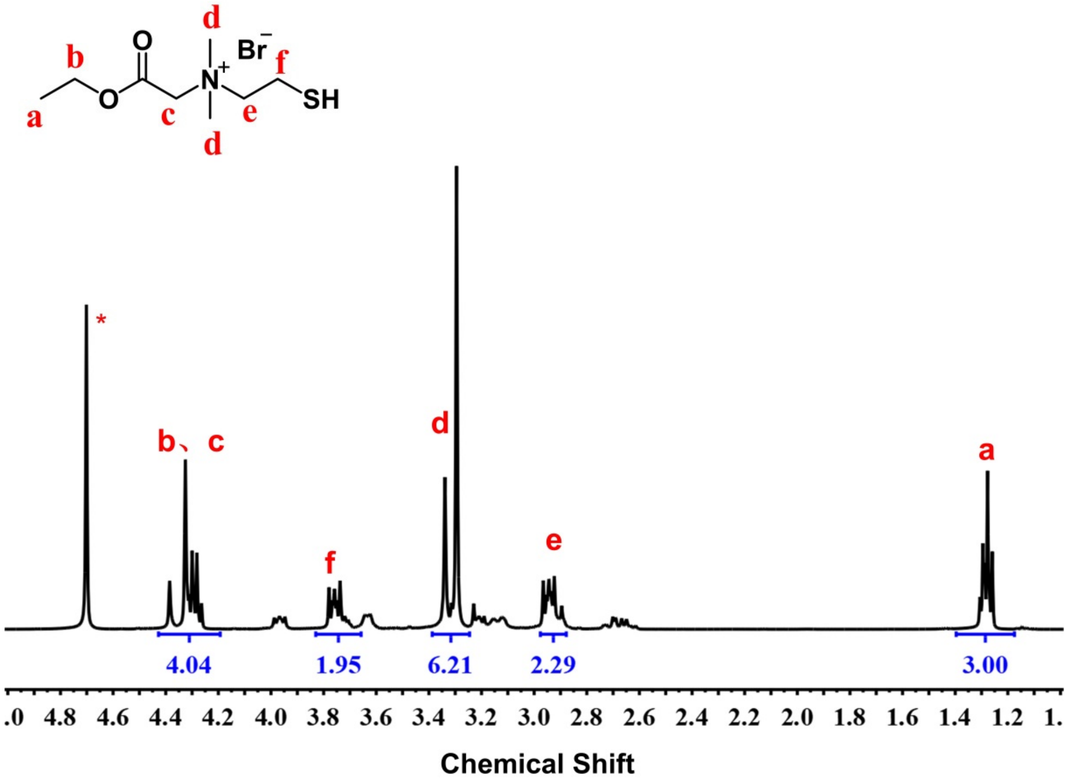

Figure S4. ^{1}H NMR spectra of 2-ethoxy-*N*-(2-mercaptoethyl)-*N*,*N*-dimethyl-2-oxoethan-1-aminium bromide. (* indicates DMSO).
